# Inferring Latent Disease-lncRNA Associations by Faster Matrix Completion on a Heterogeneous Network

**DOI:** 10.3389/fgene.2019.00769

**Published:** 2019-09-04

**Authors:** Wen Li, Shulin Wang, Junlin Xu, Guo Mao, Geng Tian, Jialiang Yang

**Affiliations:** ^1^College of Computer Science and Electronic Engineering, Hunan University, Changsha, China; ^2^Geneis Beijing Co., Ltd., Beijing, China

**Keywords:** heterogeneous bilayer network, association prediction, matrix completion, faster SVT, randomized partial SVD, similarity measurements

## Abstract

Current studies have shown that long non-coding RNAs (lncRNAs) play a crucial role in a variety of fundamental biological processes related to complex human diseases. The prediction of latent disease-lncRNA associations can help to understand the pathogenesis of complex human diseases at the level of lncRNA, which also contributes to the detection of disease biomarkers, and the diagnosis, treatment, prognosis and prevention of disease. Nevertheless, it is still a challenging and urgent task to accurately identify latent disease-lncRNA association. Discovering latent links on the basis of biological experiments is time-consuming and wasteful, necessitating the development of computational prediction models. In this study, a computational prediction model has been remodeled as a matrix completion framework of the recommendation system by completing the unknown items in the rating matrix. A novel method named faster randomized matrix completion for latent disease-lncRNA association prediction (FRMCLDA) has been proposed by virtue of improved randomized partial SVD (rSVD-BKI) on a heterogeneous bilayer network. First, the correlated data source and experimentally validated information of diseases and lncRNAs are integrated to construct a heterogeneous bilayer network. Next, the integrated heterogeneous bilayer network can be formalized as a comprehensive adjacency matrix which includes lncRNA similarity matrix, disease similarity matrix, and disease-lncRNA association matrix where the uncertain disease-lncRNA associations are referred to as blank items. Then, a matrix approximate to the original adjacency matrix has been designed with predicted scores to retrieve the blank items. The construction of the approximate matrix could be equivalently resolved by the nuclear norm minimization. Finally, a faster singular value thresholding algorithm with a randomized partial SVD combing a new sub-space reuse technique has been utilized to complete the adjacency matrix. The results of leave-one-out cross-validation (LOOCV) experiments and 5-fold cross-validation (5-fold CV) experiments on three different benchmark databases have confirmed the availability and adaptability of FRMCLDA in inferring latent relationships of disease-lncRNA pairs, and in inferring lncRNAs correlated with novel diseases without any prior interaction information. Additionally, case studies have shown that FRMCLDA is able to effectively predict latent lncRNAs correlated with three widespread malignancies: prostate cancer, colon cancer, and gastric cancer.

## Introduction

Long non-coding RNAs are RNA molecules whose transcripts are not less than 200 nucleotides, including intronic/exonic lncRNAs, antisense lncRNAs, overlapping lncRNA and long intergenic ncRNAs (lincRNAs). LncRNAs have long been considered as transcriptional noise, because of their absence in encoding proteins. Recently, it has been found that some lncRNAs regulate the expression of target genes after transcription, whose malfunction may lead to a number of diseases. For example, abnormal lncRNA expression may be involved in certain stages of cancer progression, which can serve as a potential biomarker for early tumor diagnosis (Zhou et al., 2015; Niknafs et al., 2016). In addition, lncRNAs are found able to interact with signaling pathways involved in the pathology of malignancy (Bian et al., 2015). However, studies on the prediction of relationships between lncRNAs and diseases are still limited in number. One key bottleneck is the high cost and labor-intensity of laboratory techniques in discovering the relationships between lncRNAs and diseases. To break the bottleneck, a lot of computational models have been proposed which can generally be divided into two major categories depending on the source of the interaction data: models for single-interaction data sources and models for multi-interaction data sources.

In the first major category, models for single-interaction data sources are based on diseases-lncRNAs interaction (association/link) data, which is unique known interaction information. According to its method, the model can be divided into two minor branches. The first minor branch is composed of machine-learning based models, in which the prediction of latent disease-lncRNA association takes experimentally validated disease-lncRNA associations as labeled data (training set) and unknown associations as unlabeled samples (invalidated relationship information). For example, a method named Laplacian Regularized Least Squares (LRLSLDA) was first proposed by Chen et al. to infer disease-lncRNA associations with a semi-supervised learning model (Chen et al., 2013). It is assumed that diseases with high semantic similarity are more likely to interact with lncRNAs with high functional similarity. LRLSLDA effectively predicts latent associations without negative samples, but it is difficult to select appropriate parameters and classifiers that optimize similarity measures for both lncRNAs and diseases. Inspired by the recommendation system, the authors consider disease-lncRNA association prediction as a recommendation task. A computational model named SIMCLDA is designed to predict latent disease-lncRNA relationships, taking advantage of the inductive matrix completion (IMC) method (Lu et al., 2018). The main idea of SIMCLDA is to extract informative feature vectors of lncRNAs and diseases to complete the association matrix. It is able to discover more accurate primary feature vectors and predict associations for novel lncRNAs and diseases.

Additionally, the second minor branch is composed of network-based models, random walk and a variety of propagation algorithms implemented on a heterogeneous network to infer latent disease-lncRNA associations. The heterogeneous network is constructed by integrating lncRNA-disease interaction network, disease similarity network and lncRNA similarity network. For instance, based on the hypothesis that functional lncRNAs are associated with diseases with similar phenotypes, a lncRNA functional similarity network (LFSN) is constructed and a novel computational framework RWRlncD is proposed for predicting latent disease-lncRNA associations through random walk with restart (Sun et al., 2014). However, the method of RWRlncD fails to infer related lncRNAs for novel diseases without prior interaction. Gu et al. put forward a global network-based random walk where negative samples are not required to predict latent disease-lncRNA relationships (Gu et al., 2017). Although this method can predict relationships related to isolated diseases or lncRNAs, it is prone to biased prediction. A computational method called BPLLDA is brought forward in a heterogeneous network on a basis of simple paths with finite length (Xiao et al., 2018). However, BPLLDA also has some limitations such as biased predictions. And the simplistic distance-decay function has yet to be improved by machine learning.

Due to the fact that known experimentally validated disease-lncRNA interactions are still rare, the second major category of computational models is models based on multi-interaction data sources proposed for association prediction. Multi-interaction data sources, such as lncRNA-gene interaction, lncRNAs-miRNAs interaction, disease-gene interaction and miRNA-disease interaction, are also included to infer latent disease-lncRNA associations. For example, a TPGLDA method is proposed to identify the underlying relationships by a tripartite graph of disease-lncRNA-gene and to develop an efficient resource allocation algorithm in the graph (Ding et al., 2018). TPGLDA effectively reduces the biased prediction in the resource allocation process, but it focuses on an unweighted tripartite graph and its accuracy for prediction needs to be improved. In this category, the weights of heterogeneous interaction data are difficult to determine, so the fusion of heterogeneous data is a challenging task.

Inferring latent disease-lncRNA association can also be modeled as a recommendation system which recommends top-ranked lncRNAs for given diseases. Based on matrix completion, the establishment of the disease-lncRNA recommendation system aims to complete unknown terms in the association matrix according to its ranked scores. Similar to the hypothesis in the user-item recommendation system that users with similar behaviors prefer similar items, the prediction of disease-lncRNA association assumes that phenotypically similar diseases tend to interact with functionally similar lncRNAs (Chen and Yan, 2013). In our study, we assume that semantically or functionally similar diseases tend to interact with similar lncRNAs (similar in sequence, expression profiles or function). Thus, theoretically the integration of lncRNA-lncRNA and disease-disease interaction will benefit the prediction of lncRNA-disease associations. Based on that, we proposed a computational model FRMCLDA similar to the recommendation system to infer latent associated lncRNAs for queried diseases, and solved it with faster randomized partial matrix completion (fSVT) algorithm (Feng et al., 2018). FRMCLDA consolidated disease integrated similarity network, lncRNA integrated similarity network and known disease-lncRNA interaction network to construct a heterogeneous bilayer network. Then a randomized SVD technique incorporating the block Krylov-subspace iteration (BKI) scheme (named rSVD-BKI algorithm) was proposed to complete large-scale matrix. In addition, a novel subspace reuse technique was integrated to accelerate matrix completion. Our method is based on semi-supervised machine learning, which does not need the information of negative samples. So, it generally belongs to the first category.

Our work main contributions are threefold: first, the integrated similarities for diseases and lncRNAs were properly calculated by different methods dealing with different types of data sources. The ratio of cosine similarity in integrated similarity was better determined by learning, which extracted similarity information based on known disease-lncRNA interaction. Therefore, FRMCLDA was able to offset biased predictions by similarity integration which was not entirely dependent on known interaction. Second, diseases and lncRNAs were mapped into the same network by constructing a heterogeneous bilayer network. FRMCLDA completed the disease-lncRNA interaction matrix by completing the adjacency matrix of the large-scale heterogeneous network. Thus, the similar information was included in the association prediction. Third, we took advantage of an effective fSVT algorithm which adopted rSVD-BKI and a novel subspace reuse technique to expeditiously approximate the dominant singular values and homogeneous singular vectors in an adaptive manner. Hence, the recommendation system could be extended for comprehensive adjacency matrices of heterogeneous bilayer networks.

For evaluating the performance of our method, cross validation experiments were performed on three benchmark databases, Dataset 1, Dataset 2 and Dataset 3. FRMCLDA obtained reliable AUCs of 0.92068, 0.91224 in global LOOCV and local LOOCV respectively in Dataset1, at least 5% higher than other comparison models. In Dataset 2 and Dataset 3, FRMCLDA achieved an AUC of 0.9182 and 0.8999 by global 5-fold CV, higher than other comparison methods. In addition, a case study on inferring latent lncRNAs associated with prostate cancer, colon cancer and gastric cancer in Dataset 3 were performed. In terms of the results, 16, 15, 16 out of the top 20 predicted lncRNAs associated with prostate cancer, colon cancer and gastric cancer respectively were confirmed by recent literature and public databases. The results show that FRMCLDA is able to effectively infer the associations between diseases and lncRNAs with higher accuracy than the other existing models.

## Materials and Methods

We denote a disease-lncRNA association matrix as $DL\in {\mathbb{R}}^{m\times n}$, the rows of which represent diseases and columns represent lncRNAs. The variable m is the number of diseases, and n is the number of lncRNAs. If disease *d_i_* is associated with lncRNA *l_j_*, the value of *DL*(*i*, *j)* in the association matrix is 1. And if the link between *d_i_* and *l_j_* is unknown or uncertain, *DL*(*i*, *j*) is 0. It is noted that the unlinked evidence between *d_i_* and *l_j_* is difficult to obtain. The known experimentally validated disease-lncRNA links can be retrieved from the public association database, based on which disease-lncRNA interaction matrix *DL* is established. If the number of nonzero elements is far smaller than that of zero elements, and the distribution of nonzero elements in the matrix is irregular, the matrix will be called sparse matrix. Generally, matrix *DL* is a sparse matrix, because, due to the insufficient number of studies, there are much more unknown associations (value 0) in matrix *DL* than known ones (value 1) (see **Table 1**). *LS* and *DS* denote lncRNA integrated similarity matrix and disease integrated similarity matrix respectively, which can be calculated through various biological data. It is assumed that the underlying determinants of the disease-lncRNA associations are closely related. Hence the number of independent factors is less than the number of lncRNAs or diseases. Accordingly, the matrix of known disease-lncRNA association is of low rank. That assumed, matrix completion can recover the unknown items of the disease-lncRNA interaction matrix by constructing a low-rank matrix which aims to approximates adjacency to matrix A.

**Table 1:** Details of three benchmark datasets.

Datasets	Number of known associations	Number of lncRNAs	Number of diseases	Sparsity of the matrix DL	Weights in integrated Similarity
**Dataset 1**	352	156	190	1.187*10^−2^	*w_l_* = 0.7, *w_d_* = 0.9
**Dataset 2**	540	115	178	2.638*10^−2^	*w_l_* = 0.5, *w_d_* = 0.7
**Dataset 3**	621	258	226	1.065*10^−2^	*w_l_* = 0.5, *w_d_* = 0.5

The sparsity is calculated by the ratio of existed known association number to the size of the matrix (all the possible association number).

### Overview

**Figure 1:**
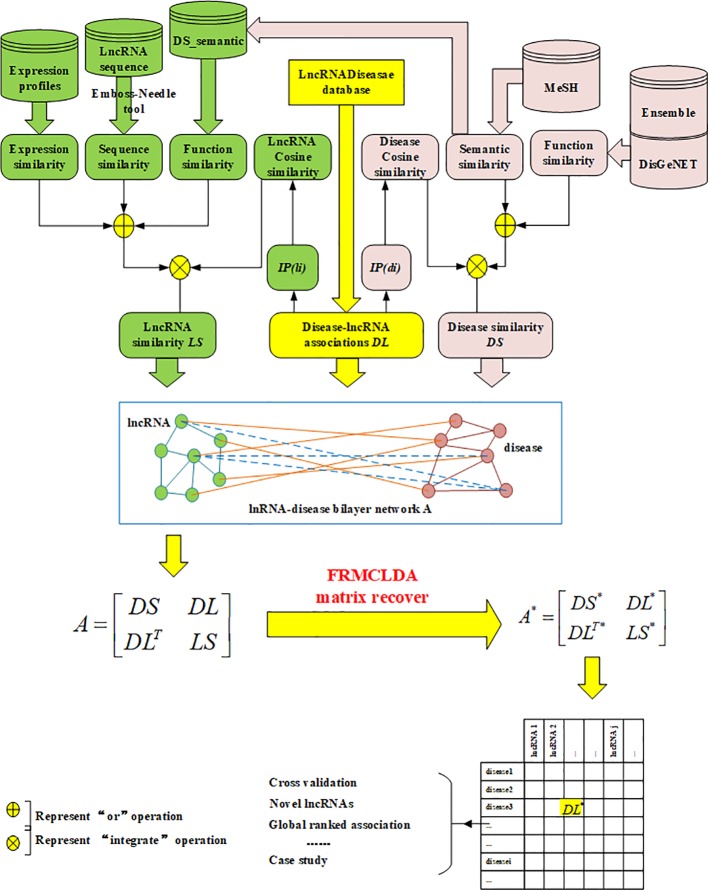
Scheme of FRMCLDA to infer latent disease-related lncRNAs by matrix recovery.

In our work, a new method called FRMCLDA is proposed to infer latent disease-lncRNA associations on the basis of fast matrix completion. The mechanism of FRMCLDA is shown in **Figure 1**. Firstly, we obtaine known disease-lncRNA associations from the public databases. Secondly, we calculate the disease similarity and lncRNA similarity with different methods. Next, we construct a heterogeneous bilayer network with three networks, i.e., a disease similarity network, a lncRNA similarity network and a disease-lncRNA interaction network. Furthermore, we implement a faster matrix completion algorithm with an improved randomized partial SVD and a sub-space reuse technique to restore the adjacency matrix of heterogeneous bilayer network. Finally, we infer potential disease-lncRNA associations through the predicted scores.

### Datasets and Data Preprocessing

All the known diseases-lncRNA interactions were obtained from three gold standard databases in three benchmark datasets respectively: MNDR database, Lnc2Cancer database and LncRNADisease database (Chen et al., 2013; Wang et al., 2013; Ning et al., 2016).

The known associations between lncRNA and disease in Dataset 1 were retrieved from the MNDR database in 2015. After removing all the duplicate records of lncRNAs and diseases, and what do not belong to human beings, and correcting the names of the lncRNAs (according to LncRNAdb, Lncipedia, NCBI and HGNC) and diseases (according to UMIS, MeSH and NCBI), we finalized 352 disease-lncRNA associations, including 156 lncRNAs and 190 diseases.

In Dataset 2, the known associations between lncRNA and disease were obtained from the Lnc2Cancer database in 2016. After eliminating the duplicate associations on account of different evidences, we obtained 540 distinct known disease-lncRNA associations, including 115 lncRNAs and 178 diseases.

In Dataset 3, the known disease-lncRNA associations were downloaded from the manually curated LncRNADisease database (http://cmbi.bjmu.edu.cn/lncrnadisease) in 2015. In the same way as data preprocessing, we downloaded 621 known disease-lncRNA associations, including 248 lncRNAs and 226 diseases. The details of the three datasets are shown in **Table 1**.

### Similarity Calculation

#### Diseases Similarity

In the three benchmark datasets, disease integrated similarities were calculated with three different similarity data sources.

a) *Disease semantic similarity:* In previous studies by Chen et al., a graph of directed acyclic (DAG) is utilized to label a disease, which includes overall relevant annotation labels acquired from the U.S. National Library of Medicine (MeSH) (Cai et al., 2008; Chen and Yan, 2013). It is assumed that diseases sharing larger common DAGs areas might have higher similarity scores. Therefore, the semantic similarity of diseases denoted as *DS*–*semantic* was calculated on the basis of DAG values by DOSim. DOSim is a package of R language for the semantic similarity calculation based on disease ontology (Wang et al., 2007).
b) *Disease functional similarity:* Disease functional similarity was calculated using the Jaccard similarity coefficient on account of gene-gene ontology relationships and disease-gene relationship, as reported in previous studies (Pinero et al., 2017; Lu et al., 2018). Disease functional similarity is denoted as *DS*–*jaccard*(*d_i_*, *d_j_*), and can be calculated by formula (1):
  (1) $$DS\_jaccard(d_{i},d_{j})=\frac{\left|GO_{d_{i}}\cap GO_{d_{j}}\right|}{\left|GO_{d_{i}}\cup GO_{d_{j}}\right|}$$
  where ***GO****_di_* represents the gene ontology terms related to disease ***d****_i_*, and the symbol |·| represents the number of items in a set.
c) *Disease cosine similarity:* Widely used in information retrieval and data mining, the cosine similarity is a popular method for calculating the similarity as the cosine of the angle between vectors. Here we used cosine similarity to extract disease feature information from the known interaction matrix *DL*. The disease cosine similarity denoted as *DS*–cos*ine*(*d_i_*, *d_j_*)can be calculated by formula (2):
  (2) $$DS\_\cos ine(d_{i},d_{j})=\frac{IP(d_{i})\cdot IP(d_{j})}{\left\|IP(d_{i})\right\|\left\|IP(d_{\text{j}})\right\|}$$
  where *IP*(*d_i_*) is the interaction profile of disease *d_i_*, the *i-th* row vector of the interaction matrix *DL*. If disease *d_i_* is associated with lncRNA *l_k_*, the k-th element in *IP*(*d_i_*) is 1, otherwise the value is 0. The value 0 does not mean that association does not exist but means it is uncertain. ||*IP*(*d_i_*)|| is the 2-norm of *IP*(*d_i_*).
d) *Integrated disease similarity DS:* To illustrate the adaptability of our model to different similarity data, we adopted two different integrated disease similarities in three benchmark datasets. In Dataset 1 and Dataset 2, *DS* was calculated by: $DS=w_{d\text{1}}*DS\_semantic+(1-w_{d\text{1}})*DS\_\cos ine$ In Dataset 3, *DS* was calculated by: $DS=w_{d\text{2}}*DS\_jaccard+(1\text{-}w_{d\text{2}})*DS\_\cos ine$.

#### LncRNAs Similarity 

To calculate lncRNA integrated similarity, we adopted four different sources of similarity data: lncRNA sequence similarity, lncRNA expression similarity, lncRNA functional similarity and lncRNA cosine similarity.

a) *LncRNA sequence similarity:* Most of the RNA sequences of lncRNAs were downloaded mainly from the database LncRNADisease (http://www.cuilab.cn/lncrnadisease). The sequences not available in LncRNADisease were retrieved from the databases UCSC and LNCipedia. The sequence similarity between two lncRNAs were calculated with Needleman-Wunsch global alignment algorithm (Emboss-Needle tool) (Needleman and Wunsch, 1970; Rose and Eisenmenger, 1991). We set the parameters to default values. The Matrix file name was set to EDNAfull for nucleic, Gap opening penalty was set to 10 and Gap extension penalty was set to 0.5 for any sequences. LncRNA sequence similarity is defined as formula (3):
  (3) $$LS\_seq(l_{i},l_{j})=\frac{SW(l_{i},l_{j})}{\sqrt{SW(l_{i},l_{i})\cdot SW(l_{j},l_{j})}}$$
  where *SW*(*l_i_*, *l_j_*) is the alignment score calculated by Emboss-Needle, which is equal to the sum of the matches taken from the scoring matrix, minus penalties arising from opening and extending gaps in the aligned sequences.
b) *LncRNA expression similarity:* The expression profiles of lncRNA can be obtained from the dataset E-MTAB-513 in ArrayExpress (Parkinson et al., 2007; Derrien et al., 2012). Based on the previous literature, we normalized these expression data and calculated the lncRNA expression similarity *LS*_exp with the absolute Spearman correlation coefficient (Chen and Yan, 2013).
c) *LncRNA functional similarity:* Based on an accepted assumption that lncRNAs with similar functions have similar interaction patterns to those of diseases, the functional similarity of lncRNA can be obtained *via* computation of disease semantic similarity from a previous study by Sun et al., (2014). It is supposed that lncRNA *l_i_* is correlated with a set of diseases *D_i_* = {*d_i_*_1_, *d_i_*_2_,…, d_im_}, and lncRNA *l_j_* is correlated with a set of diseases *Dj* = {*d_j_*_1_, *d_j_*_2_,…*d_jn_*}. Semantic similarity between *d_il_* and *Dj* is calculated as formula (4):
  (4) $$DS\_semantic(d_{il},D_{j})=\underset{d\in D_{j}}{\max}(DS\_semantic(d_{il},d))$$
  And then the functional similarity of lncRNAs can be computed as formula (5):
  (5) LS_func(li,lj)=∑1≤l≤mDS_semantic(dil,Dj)+∑1≤k≤nDS_semantic(djk,Di)m+n
  where *LS*_*func*(*l_i_*, *l_j_*) denotes the functional similarity between lncRNA *l_i_* and lncRNA *l_j_*.
d) *LncRNA cosine similarity:* In the same way, we used cosine similarity to extract lncRNA feature information from the known interaction matrix *DL*. lncRNA cosine similarity can be calculated as formula (6):
  (6) $$LS\_\cos ine(l_{i},l_{j})=\frac{IP(l_{i})\cdot IP(l_{j})}{\left\|IP(l_{i})\right\|\left\|IP(l_{j})\right\|}$$
  where *IP*(*l_j_*) is resulted from the *j-th* column of the interaction matrix *DL*. *IP*(*l_j_*) is a vector which denotes the feature vector for lncRNA *l_j_*.
e) *Integrated lncRNA similarity LS:* In three benchmark datasets, we adopted three different similarity computation methods to fully demonstrate the robustness of FRMCLDA. In Dataset 1, integrated lncRNA similarity *LS* was calculated as: $LS=w_{l\text{1}}*LS\_func+(1-w_{l\text{1}})*LS\_\cos ine$. In Dataset 2, *LS* was calculated as: $LS=w_{l\text{2}}*LS\_\exp+(1-w_{l\text{2}})*LS\_\cos ine$. In Dataset 3, *LS* was calculated as: $LS=w_{l\text{3}}*LS\_seq+(1-w_{l\text{3}})*LS\_\cos ine$.

### Construction of the Heterogeneous Bilayer Network

Based on the integrated disease and lncRNA similarity matrices *DS* and *LS* calculated above, disease similarity network and lncRNA similarity network can be constructed. Let *D* = {*d*_1_, *d*_2_,…, *d_n_*} represent the set of n diseases in the disease similarity network. The edge between disease *d_i_* and *d_j_* is weighted by integrated disease similarity *DS*(*i*, *j*). Let *L* = {*l*_1_, l_2_,…, *l_m_*} represent the set of m lncRNAs in the lncRNA similarity network. The edge between lncRNA *l_i_* and *l_j_* is weighted by integrated lncRNA similarity *LS*(*i*, *j*). Besides, the disease-lncRNA interaction network can be modeled as *G*(*V*, *E*), where *V*(*G*) = {*D*,*L*}, *E*(*G*) ⊆ *D* × *L*, *E*(*G*) = {*e_ij_*, edge between disease *d_i_* and lncRNA *l_j_*}. The edge *e_ij_* is initialized to 1, if there exists a known link between disease *d_i_* and lncRNA *l_j_*, otherwise, *e_ij_* is initialized to 0. *DL* is the adjacency matrix for the disease-lncRNA interaction network.

Finally, a heterogeneous bilayer network is constructed by connecting disease similarity network and lncRNA similarity network *via* disease-lncRNA association network, as shown in **Figure 1**. Accordingly, the adjacency matrix A of the heterogeneous bilayer network is defined as formula (7):

(7) $$A=\left[\begin{array}{cc} DS & DL\\ DL^{T} & LS \end{array}\right]$$

where diagonal sub-matrices *DS* and *LS* are the adjacency matrix of the disease similarity network and the lncRNA similarity network. The off-diagonal sub-matrix *DL* is the adjacency matrix for the disease-lncRNA interaction network, *DL^T^* is the transpose of *DL*. Usually, the interaction between lncRNAs and diseases is mutual, and values of the matrix *DL* are nonnegative, therefore the adjacent matrix A is meristic and positive semi-definite. The singular values of the adjacent matrix A are nonnegative real numbers and equivalent to the eigenvalues. In conclusion, the prediction of disease-lncRNA association can be remodeled as the matrix completion of the adjacency matrix A. If matrix A is only comprised of matrix *DL*, rather than the large-scale matrix of the heterogeneous network, then the completion based on rank minimization will not generate significant results. That is because all known disease-lncRNA associations are positive in matrix. Only restoring the matrix *DL* will result in an optimized solution to rank minimization problem, i.e., all-one matrix with rank 1.

### Inferring Latent Associations by Faster Randomized Matrix Completion

Our goal is to restore the unknown entries of the adjacent matrix A by constructing a proximate matrix A^*^ with the same size (*m* + *n*) × (*m* + *n*). It is assumed that A have rank *r*(*r* ≪ (*m* + *n*)). Φ is denoted as an index set for all the known entries of matrix A. The problem of matrix completion can be converted to solving the rank minimization problem by formula (8):

$$\min rank(A^{*})$$

(8) $$s.t.P_{\Phi }(A^{*})=P_{\Phi }(A)$$

in which *P*_Φ_(*A*) is denoted as an orthogonal projector onto the span of matrix A. Its value is 0 when the element (*i*, *j*) is not in the set Φ. Matrix *A* is the adjacency matrix of the heterogeneous network constructed in *Datasets and Data Processing*. However, the problem of rank minimization is generally considered as a NP-hard problem (Natarajan, 1995). An approach called relaxed convex optimization is widely used by minimizing the nuclear norm (||·||_*_) of the matrix, which is known to be solved by standard singular value threshold (SVT) algorithm (Candès and Recht, 2009). Therefore, the matrix completion can be resolved by a proximal optimization solution (Cai et al., 2008). Minimization of the nuclear norm can be resolved by formula (9):

$$\min{\left\|A^{*}\right\|}_{*}$$

(9) $$s.t.P_{\Phi }(A^{*})=P_{\Phi }(A)$$

Equation (9) can be solved by the iterative processes in formula (10) and (11):

(10) $$\{\begin{array}{c} X^{\left(i\right)}=shrink(Y^{\left(i-1\right)},\tau )\\ Y^{\left(i\right)}=Y^{\left(i-1\right)}+\delta P_{\Phi }(A-X^{\left(i\right)}) \end{array}$$

(11) $$shrink(Y^{\left(i\right)},\tau )=\sum_{j=1}^{\sigma_{j}^{\left(i\right)}\geq \tau }\left(\sigma_{j}^{\left(i\right)}-\tau \right)u_{j}^{\left(i\right)}v_{j}^{(}$$

where the function *shrink*(*Y*^(^*^i^*^)^, τ) is a soft thresholding operator that computes the singular value of the matrix *Y* at level τ (Aken et al., 2016). δ is the iteration step length. $\sigma_{j}^{\left(i\right)}$ is one of the singular values of *Y*at the *i*th iteration. $u_{j}^{\left(i\right)}$ and $v_{j}^{\left(i\right)}$ are the corresponding left singular vector and right singular vector respectively. *Y*^(^*^i^*^)^ is usually a relatively large matrix with high sparsity, and usually can be stored with a sparse matrix. Starting computation from $Y^{\text{(0)}}\text{=c}\delta P_{\Phi }\text{(A)}$, a series of *X*^(^*^i^*^)^ and *Y*^(^*^i^*^)^ can be generated through the linearized Bregman iteration.

In FRMCLDA, δ is set to $(\text{m}+\text{n)}/\sqrt{\left|\Phi \right|}$ as assigned in previous literature (Li and Yu, 2017). We set $c=\left\lceil \tau /(\delta \left\|p_{\Phi }(A)\right\|)\right\rceil$, $\tau \;=\;∥P_{\phi }(A){∥}_{F}(m\;+\;n)/\sqrt{\left|\Phi \right|}$ to balance the accuracy of approximation against the speed of convergence as suggested by Candès et al.(Candès and Recht, 2009). Although the SVT algorithm has high accuracy for both symmetric and real data matrix, the costs are large when executing SVD repeatedly at each iteration. So many improved methods like truncated singular value decomposition and randomized SVD have been proposed for accelerating SVT by keeping the cost of shrink (·) low throughout the iteration (Halko et al., 2010). In this study, FRMCLDA adopts a faster SVT algorithm, fSVT, based on partial and improved randomized SVD which exploits a sub-space reuse technique to extract key singular value and corresponding singular vector. The main concept of randomization is to determine the sub-spaces for obtaining dominant information and ignore insignificant information by random projection. Randomized SVD algorithms execute as many or fewer floating-point operations (*flops*) with the runtime benefit. Faster matrix completion even incorporates a block Krylov sub-space iteration rSVD-BKIr scheme and a novel sub-space reuse mechanism (reuse the orthogonal basis Q from the last round of iteration) (Musco and Musco, 2015). The fast matrix completion algorithm with rSVD-BKI has been proven the have the same reliability and accuracy as the original singular value thresholding algorithm, while at higher speed for large data matrix completion (Feng et al., 2018). FRMCLDA applies the faster singular value threshold (fSVT) algorithm for a similarly optimal low-rank approximation of the adjacency matrix, and prediction of latent links between lncRNAs and diseases in *LD*. Our faster randomized matrix completion method is illustrated in Algorithm 1 in the **Supplementary Materials S6**. The function *rSVD*-*BKI*(·) performs singular value decomposition and the details of realization can be found in an earlier study (Feng et al., 2018)

## Experiments and Results

We first put forward the evaluation metrics for the methods of association prediction. Second, we tested the effects of cosine similarity on diseases and lncRNAs and fine-tuned the weights of cosine similarity. Third, we implemented permutation test to assess the influence of different data sources on optimization procedure. Fourth, we recorded the time usage of FRMCLDA for different sizes of heterogeneous network. Fifth, we compared FRMCLDA with other existing methods by global LOOCV experiments, local LOOCV experiments and global 5-fold cross-validation experiments. Finally, we implemented case studies to validate the practicability of FRMCLDA.

### Evaluation Metrics of Performance

In order to assess the performance of FRMCLDA in inferring latent disease-correlated lncRNAs, global LOOCV experiments, local LOOCV experiments and global 5-fold cross-validation experiments are implemented on three benchmark datasets. Under the framework of LOOCV, each known experimentally validated association is picked in turns as a test sample, and all the other known associations are considered as training samples. The test sample is sorted together with the candidate samples without known association evidence. The test sample whose rank exceed the given threshold would be considered as a successful prediction. The main difference between global and local LOOCV is whether to investigate all diseases simultaneously or only query one disease at a time to select candidate samples. That is to say, global LOOCV considered all the unknown associations as candidate samples, whereas local LOOCV only focused on one disease in the test sample and selected the corresponding unknown associations as candidate samples. In global 5-fold cross-validation, all of the known experimentally validated associations are divided into five uncrossed sets, whose size must be strictly equal. Each set of the five is taken in turns as the test sample, but the other 4 sets are served as training samples. After matrix completion is performed, the test samples are ranked together with candidate samples and then are sorted in the descending order of their predicted scores.

Furthermore, false negative (FN), false positive (FP), true negative (TN) and true positive (TP) are summarized based on the ranked results for each specific threshold. The receiver operating characteristic curves are made by plotting the true positive rate (TPR, recall) against false positive rate (FPR) based on varying thresholds. The precision-recall (PR) curve is also plotted to fully evaluate the performance of the prediction. The area under the ROC curve (AUC) and the area under the PR curve (AUPR) are finally calculated to evaluate the overall performance of the prediction. An AUC value of 0.5 implies a random prediction and an AUC value of 1 implies a perfect prediction performance. Therefore, AUC and AUPR are used as primary evaluating measures.

### Effects of Cosine Similarity on Diseases and lncRNAs

Both integrated disease similarity and integrated lncRNA similarity in three benchmark datasets are calculated with cosine similarity combined, which can extract feature information from the known interaction matrix. The weights of *w_l_* and *w_d_* in integrated similarity calculations can be fine-tuned by cross validation in three benchmark datasets separately. Let *w_d_* and *w_l_* vary from 0.1 to 1 at the increment of 0.1. According to AUC values of LOOCV based on Dataset1, FRMCLDA performed best when *w_d_*_1_ = 0.9 and *w_l_*_1_ = 0.7. Likewise, on Dataset2, we chose *w_d_*_2_ = 0.7 and *w_l_*_2_ = 0.5. On Dataset3, we chose *w_d_*_3_ = 0.5 and *w_l_*_3_ = 0.5. All can be seen in **Table 1**.

In Dataset2, we implemented 5-fold cross validation 20 times to test the effects of the cosine similarity on model performance. The four test settings were: 1) using cosine similarity both for integrated similarity of neither lncRNAs nor diseases; 2) only the lncRNA similarity integrating the cosine similarity; 3) only the disease similarity integrating the cosine similarity; 4) both lncRNA similarity and disease similarity integrating cosine similarity. The results can be seen in **Table 2**. When both similarities are calculated with cosine similarity combined, the AUC value (0.9145 ± 0.0013) achieves the best of four. Therefore, FRMCLDA performance can be improved by incorporating effective feature information extracted by cosine similarity from interaction profiles with fast matrix completion.

**Table 2:** The effects of the cosine similarity on AUC by 5CV in dataset2.

No *LS*_cosine and *DS*_cosine	Only combing *LS*_cos*ine* in LS	Only combing *DS*_cos*ine* in DS	Combing *LS*_cos*ine* in LS and *DS*_cos*ine* in DS
0.7995 ± 0.0044	0.8705 ± 0.0050	0.8510 ± 0.0032	0.9145 ± 0.0013

### Influence of Different Data Sources on Optimization Procedure

To evaluate the influence of different data sources on the optimization procedure of matrix completion, we have implemented a permutation test on disease-lncRNA interaction matrix DL, lncRNA similarity matrix LS, and disease similarity matrix DS separately. Based on the LOOCV framework, we randomized each of the three matrices in turns, while keeping the other two matrices unchanged. We carried it out 20 times and recorded the average AUC value for each type of data source. Usually, if a matrix contributes more to the optimization procedure, the result of the permutation test based on it will be closer to the stochastic value. As shown in **Table 3**, the mean AUC based on randomized matrix DL is the lowest and close to 0.5, indicating that matrix DL has the greatest influence on the performance of our model. In the same way, it is concluded that matrix LS contributes more than DS to the performance of our model.

**Table 3:** The result of contribution test on performance of prediction by LOOCV in dataset 2.

Set the data source to random matrix	Average AUCs by 20 times randomization
lncRNA similarity matrix (LS)	0.8615 ± 0.0061
Disease similarity matrix (DS)	0.8081 ± 0.0059
disease-lncRNA association matrix (DL)	0.5332 ± 0.0174

### Time Usage of FRMCLDA for Different Sizes of the Heterogeneous Network

In FRMCLDA model, we implement matrix completion through fSVT as proposed by a previous study (Feng et al., 2018). Algorithm fSVT used a block Krylov iteration approximation SVD method rSVD-BKIr and a sub-space reuse mechanism to replace the original exact SVD. Thus, the turn-around time of SVT is significantly reduced while the accuracy remains the same. As seen in algorithm 1 in **Supplementary file S6**, tolerance ε is the terminating condition. When mean absolute error (MAE) is greater than ε, the program will terminate. The value of power parameter p can be dynamically adjusted as the operation accuracy changes. Therefore, the parameter ε and p can decide the rounds of the iteration, which will greatly affect the main turn-around time of FRMCLDA. Therefore, we were not able to compare the running time with other of other methods because of different conditions. We just recorded the time usage of FRMCLDA for different sizes of heterogeneous network.

Here, we set *p* = 2 and ε = 0.4. We executed 20 times FRMCLDA in three benchmark datasets. Average time usage of FRMCLDA and standard deviations are shown in **Table 4**. In Dataset 1, 2 and 3, the CPU time reached 2.1758 ± 0.2826 s, 1.5367 ± 0.1799 s and 3.9016 ± 0.2703 s, respectively.

**Table 4:** The time usage of FRMCLDA for different sizes of heterogeneous network.

	The size of heterogeneous network	CPU time (second)
**Dataset1**	156 × 190	2.1758 ± 0.2826
**Dataset2**	115 × 178	1.5367 ± 0.1799
**Dataset3**	258 ×226	3.9016 ± 0.2703

### Comparison of Performance With Other Methods on Different Datasets

**Figure 2:**
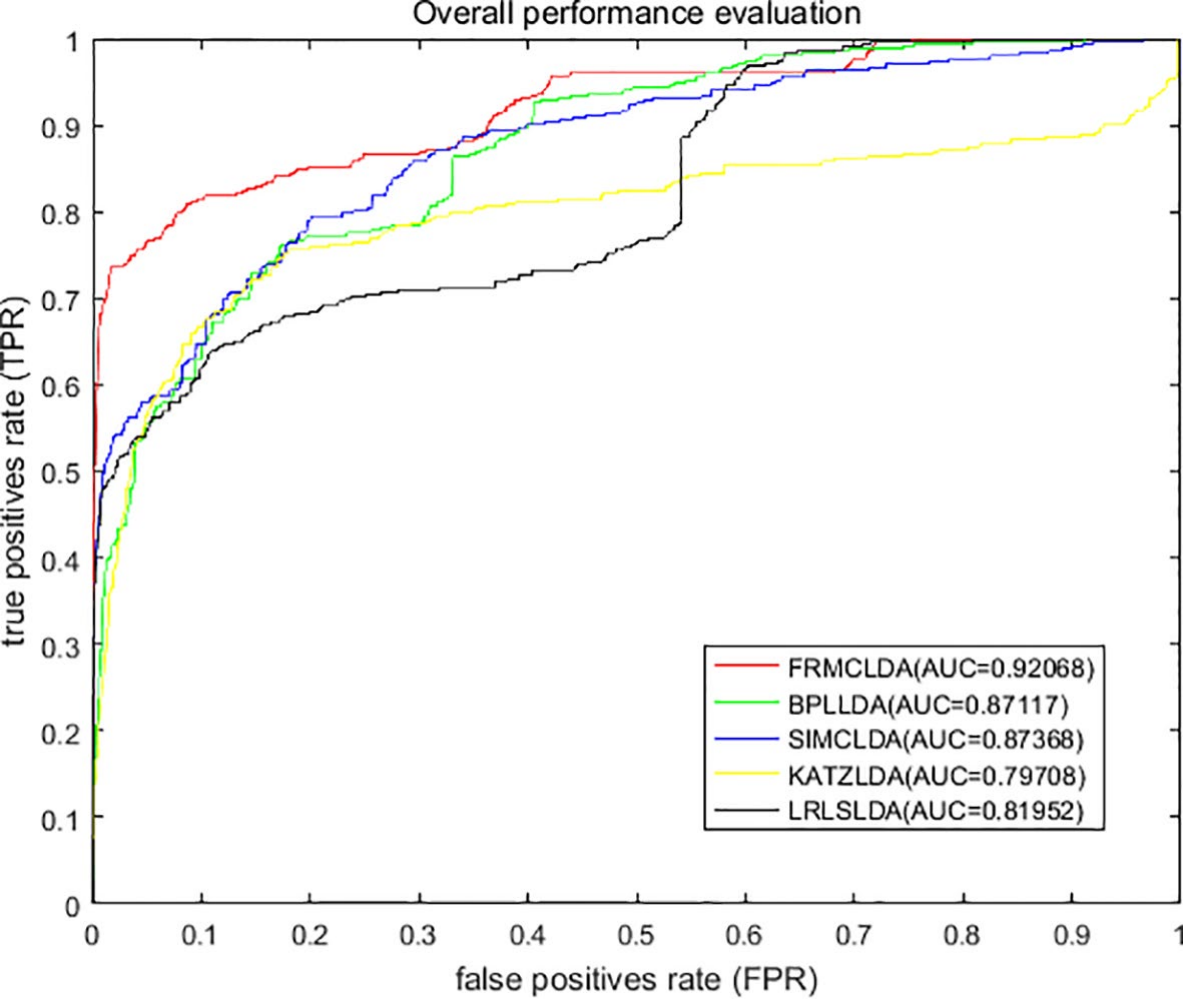
Overall performance assessment of FRMCLDA, BPLLDA, SIMCLDA, KATZLDA and LRLSLDA in predicting disease-lncRNA relationships on Dataset 1 by global LOOCV.

On Dataset 1, the performance of FRMCLDA is compared with four popular methods: LRLSLDA (Chen and Yan, 2013), KATZLDA (Chen, 2015), SIMCLDA (Lu et al., 2018) and BPLLDA (Xiao et al., 2018). The ROC curves of global LOOCV are shown in **Figure 2**. Obviously, FRMCLDA achieved an AUC of 0.92068, which outperformed LRLSLDA (0.81952), KATZLDA (0.79708), SIMCLDA (0.87368) and BPLLDA (0.87117) by 5% at least. Therefore, FRMCLDA is superior compared to other methods in predicting disease-lncRNA association.

**Figure 3:**
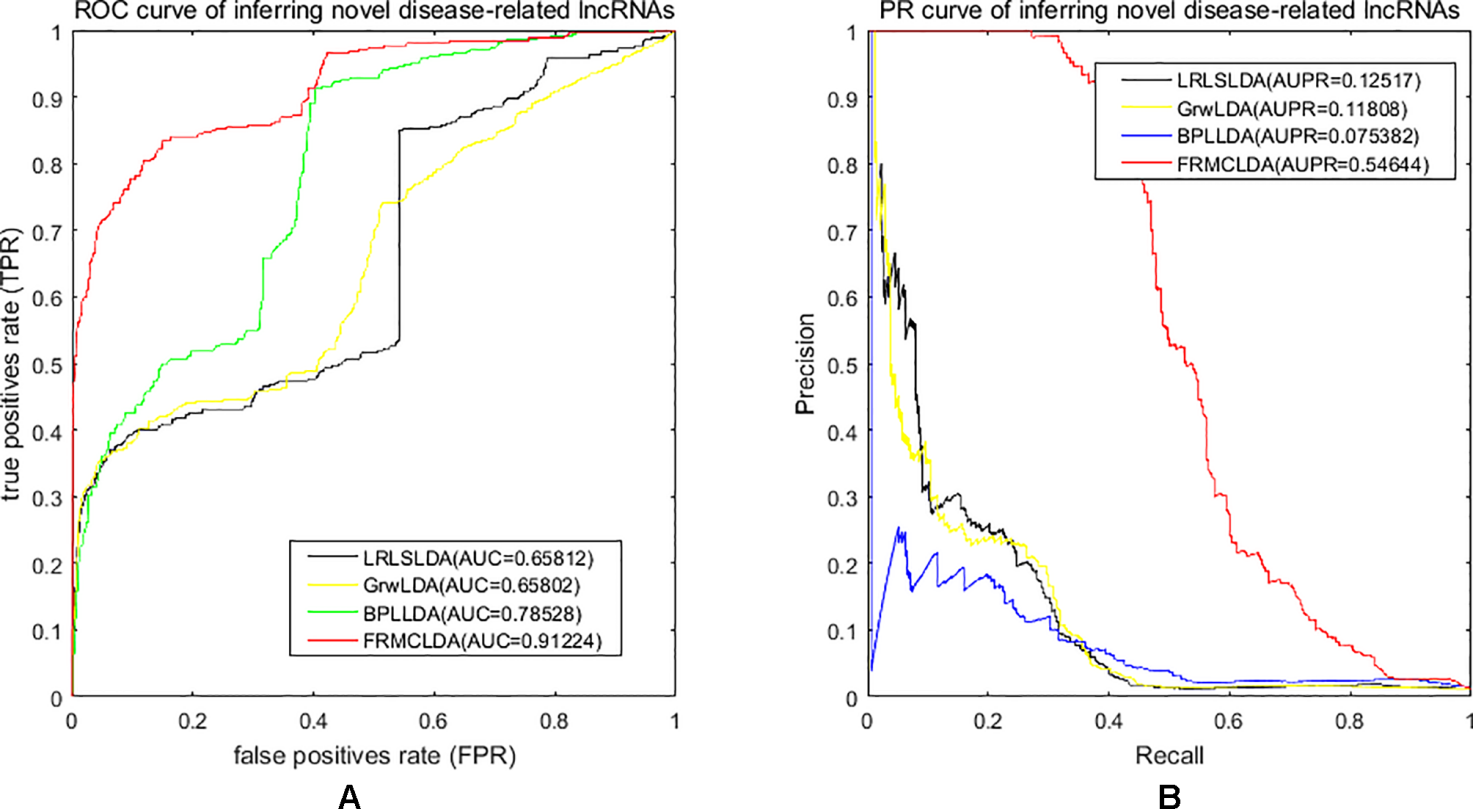
Performance assessment of LRLSLDA, GrwLDA, BPLLDA and FRMCLDA in inferring novel disease-correlated lncRNAs on Dataset 1 by local LOOCV. **(A)** ROC curve of inferring novel disease-related lncRNAs. **(B)** PR curve of inferring novel disease-related lncRNAs.

One advantage of FRMCLDA is that it is able to infer latent correlated lncRNAs with queried diseases, even novel diseases. To show the performance of FRMCLDA in predicting novel disease-correlated lncRNAs, we implemented local LOOCV on Dataset 1. The results of FRMCLDA with local LOOCV on Dataset 1 were recorded in **Supplementary S1**. As shown in **Figure 3**, compared with three methods (LRLSLDA, BPLLDA, and GrwLDA) (Gu et al., 2017), the AUC of FRMCLDA was 0.91224, significantly higher than those of LRLSLDA (0.65812), BPLLDA (0.78528) and GrwLDA (0.65802) with increases of about 27.8%, 13.9%, and 27.86% respectively. The AUPR of FRMCLDA was 0.54644, significantly higher than those of LRLASLDA (0.12517), GrwLDA (0.1180) and BPLLDA (0.0753). In conclusion, FRMCLDA has been proven to be effective in inferring related lncRNAs with novel diseases in terms of AUC values and AUPR values. For example, we deleted all the known breast cancer-correlated associations, just as breast cancer was a novel disease. After matrix completion by FRMCLDA, we ranked all the candidate lncRNAs according to their scores. As can be seen in **Table 5**, all 14 deleted breast cancer-associated lncRNAs were finally successfully ranked out of top 20 of all the predicted lncRNAs.

**Table 5:** Predicting novel disease-related lncRNAs by deleting known associations for each disease.

Known but deleted breast cancer-related lncRNAs	Rank number	Known but deleted breast cancer-related lncRNAs	Rank number
BCAR4	13	LSINCT5	7
BCYRN1	6	MALAT1	2
CDKN2B-AS1	4	MEG3	3
DSCAM-AS1	8	MIR31HG	14
GAS5	10	PINC	15
H19	1	PVT1	5
HOTAIR	9	SRA1	11

**Figure 4:**
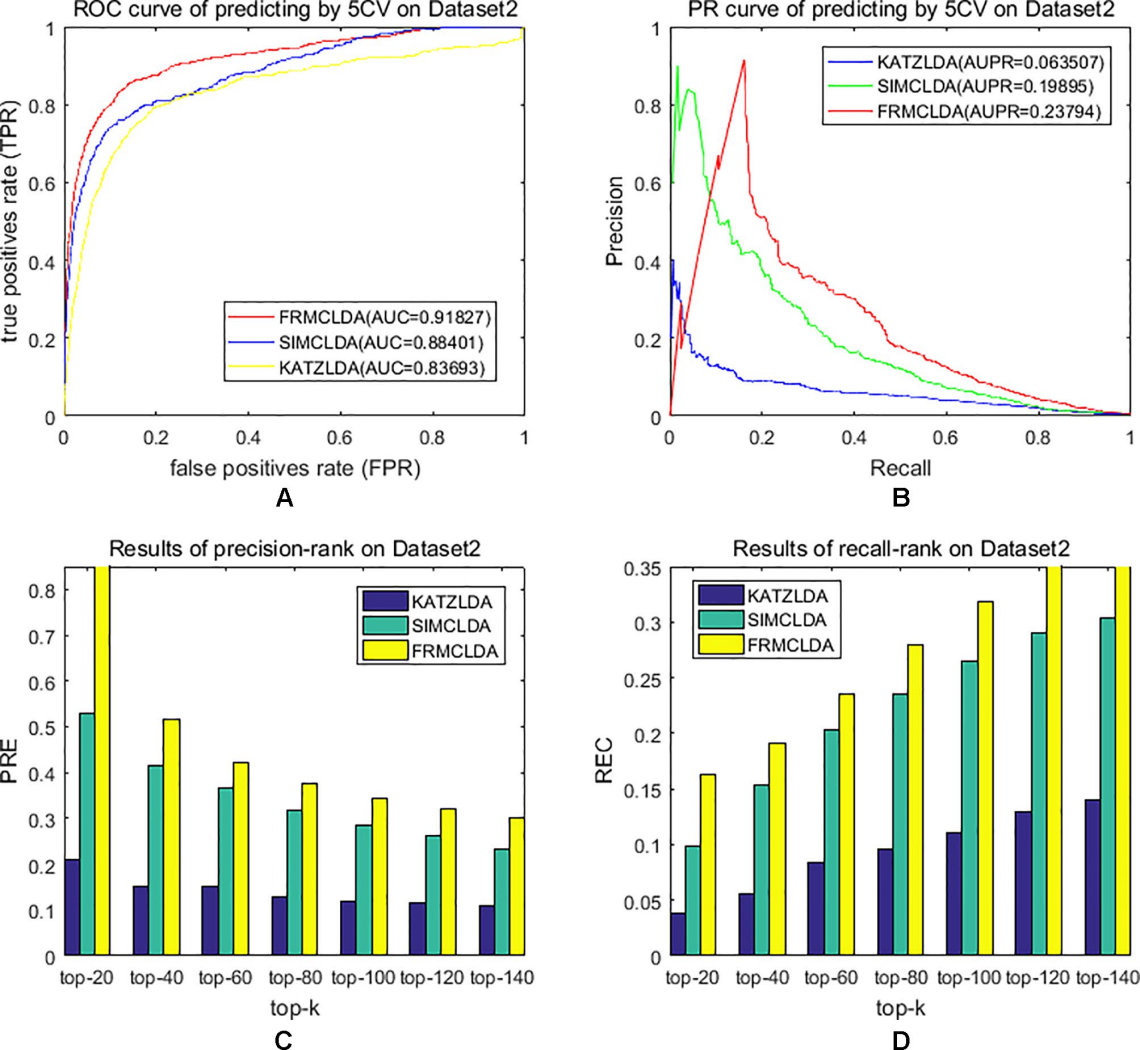
Performance of FRMCLDA, KATZLDA and SIMCLDA on inferring lncRNAs by global 5-fold cross-validation on Dataset 2. **(A)** ROC curve of predicting disease-lncRNA associations. **(B)** PR curve of predicting disease-lncRNA associations. **(C)** Results of precision at every rank. **(D)** Results of recall at every rank.

**Figure 5:**
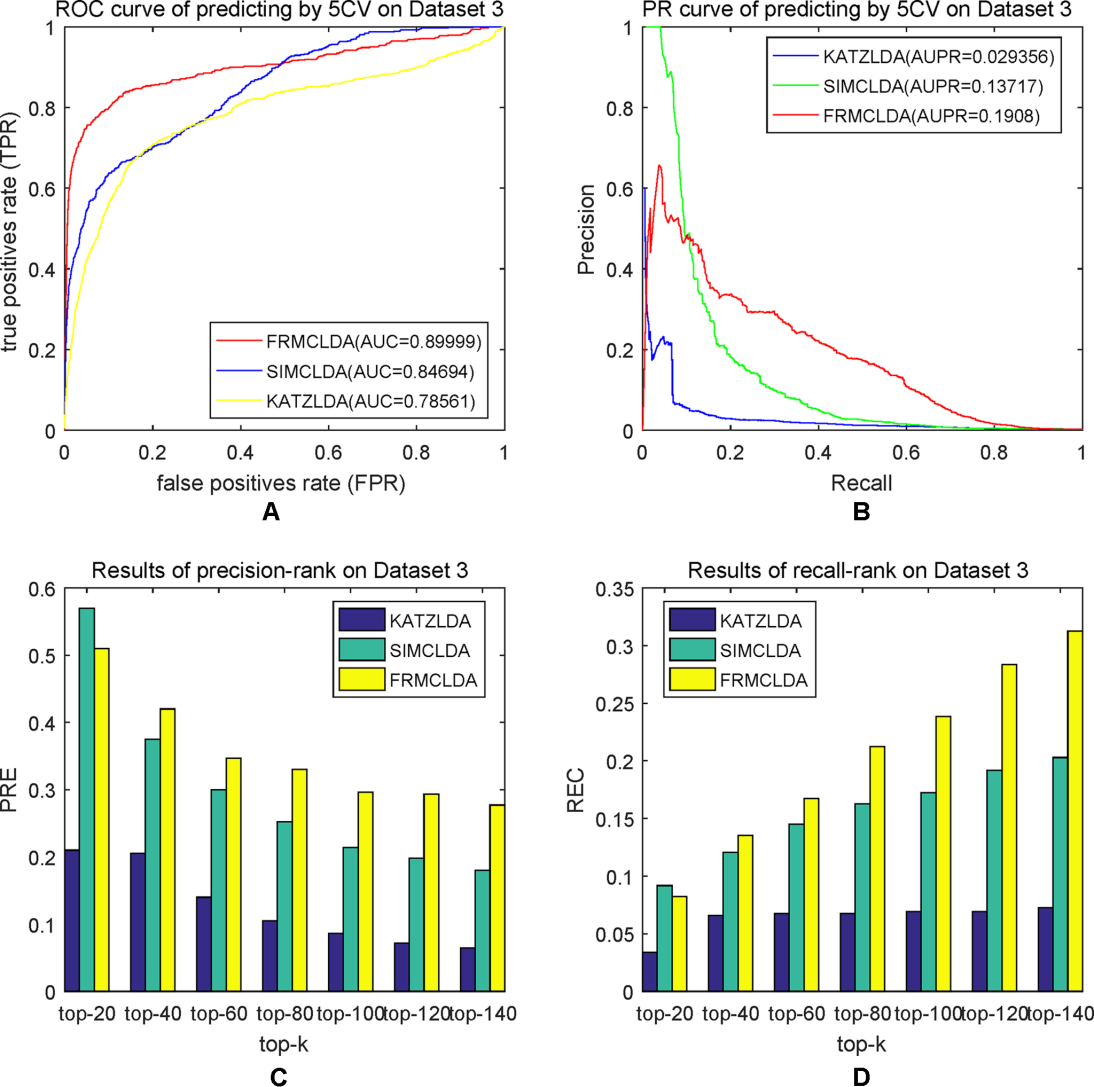
Performance of FRMCLDA, KATZLDA and SIMCLDA on inferring lncRNAs by global 5-fold cross-validation on Dataset 3. **(A)** ROC curve of predicting disease-lncRNA associations. **(B)** PR curve of predicting disease-lncRNA associations. **(C)** Results of precision at every rank. **(D)** Results of recall at every rank.

The robustness of FRMCLDA was further validated by inferring latent associations on Dataset 2 and Dataset 3. We conducted 20 times global 5-fold cross-validation experiments to validate the precision of prediction by FRMCLDA on Dataset 2 and Dataset 3. The results of ROC curve, PR curve, precision-rank bars and recall-rank bars using different methods are shown in **Figure 4** and **Figure 5**, respectively. For example, as shown in **Figure 4**, after one time global 5-fold cross-validation on Dataset 2, FRMCLDA achieved an AUC of 0.91827, higher than SIMCLDA (0.88401) and KATZLDA (0.83693). The AUPR of FRMCLDA was 0.23794, also higher than those of SIMCLDA (0.1989) and KATZLDA (0.0635). Furthermore, on Dataset 2, the maximum precision reached by FRMCLDA is 0.88, which is higher than other methods, as shown in **Table 6**. On Dataset 3, after 20 times 5-fold CV, the average AUC of FRMCLDA is 0.8999 (က±0.0049), which is superior to SIMCLDA 0.84694 (က±0.0033) and KATZLDA 0.78561 (က±0.0053). The AUPR of FRMCLDA is 0.1908 (က±0.0033), higher than those of SIMCLDA 0.13717 (က±0.0027) and KATZLDA 0.0293 (±0.0036), as shown in **Figure 5**. Furthermore, in terms of precision-rank bar and recall-rank bar, FRMCLDA boasts the best precision at every rank except for the top-20 rank in Dataset 3. In summary, FRMCLDA demonstrates high prediction accuracy on three different datasets.

**Table 6:** Precision-rank on dataset 2.

	lncRNA	Top 20	Top 40	Top 60	Top 80	Top 100	Top 120	Top 140
**precision**	FRMCLDA	0.8800	0.5150	0.4233	0.3775	0.3440	0.3200	0.3029
	SIMCLDA	0.5300	0.4150	0.3667	0.3175	0.2860	0.2671	0.2343
	KATZLDA	0.2100	0.1500	0.1500	0.1300	0.1200	0.1167	0.1086
**recall**	FRMCLDA	0.1630	0.1707	0.2352	0.2796	0.3185	0.3556	0.3926
	SIMCLDA	0.0981	0.1537	0.2037	0.2352	0.2648	0.2907	0.3037
	KATZLDA	0.0389	0.0556	0.0833	0.0963	0.1111	0.1296	0.1407

### Case Study

After performing cross validation to confirm the ability of FRMCLDA, we conducted a global prediction of potential related disease-lncRNA pairs. All known lncRNAs-diseases links were considered as training samples, while other unknown associations constituted the candidate samples. FRMCLDA can infer the latent correlated lncRNAs for all diseases simultaneously by faster random matrix completion. All candidate lncRNAs correlated with a queried disease were ranked according to predicted scores generated by FRMCLDA. The predicted and ranked lncRNAs (excluding known correlated lncRNAs) correlated with 226 diseases on Dataset 3 can be seen in **Supplementary S2**. To confirm whether top-ranked lncRNAs for queried diseases are real through public literature and three public databases (LncRNADisease, Lnc2Cancer and MNDR), we have conducted case studies on Dataset 3. Three databases are kept updated with new disease-lncRNA links verified by biological experiments in support of our validation. As shown by one of the prediction results in **Supplementary S2**, we take prostate cancer, colon cancer and gastric cancer and show the verification of top-20 predicted lncRNAs for each selected cancer.

Prostate cancer is one of the most common malignant tumors for males, accounting for about 13% of cancer-related death (Miller et al., 2016). In prostate cancer, the expression level of lncRNAs may be increasing or decreasing steadily (Smolle et al., 2017). Thus, it is justifiable to predict the possible links between lncRNAs and prostate cancer. Recent biological experiments have identified several lncRNAs associated with prostate cancer. For example, LncRNA H19 is down-regulated significantly in the cell line M12 of metastatic prostate carcinoma (Zhu et al., 2014). HOTAIR is found to be significantly regulated *via* genistein, and the expression of HOTAIR in castration-resistant PCa cell line is higher than that of standard prostate cell lines (Chiyomaru et al., 2013). MEG3 can enhance Bax, activate caspase 3 and inhibit the internal survival pathway of cells *in vivo* and *in vitro* through decreasing the bcl-2 protein expression (Zhang et al., 2003). MALAT1 is upregulated in prostate tumor tissue and cell line of human beings (Ren et al., 2013). It is reported that CBX7 and CDKN2B-AS1 levels are enhanced in prostate tumor tissues (Yap et al., 2010). PVT1 can accelerate the intrusion and transfer by prostate carcinoma *via* regulating EMT (Chang et al., 2018). Linc00963 is a new lncRNA which is involved in the transformation from the androgen-dependent stage to the androgen-independent stage of prostate carcinoma (Wang et al., 2014). In our work, FRMCLDA is performed to infer possible lncRNAs correlated with prostate cancer. Finally, 10 out of top-10 and 16 out of top-20 predicted prostate cancer-associated lncRNAs are verified on the three databases (LncRNADisease, MNDR, Lnc2Cancer) mentioned above. They are shown in **Table 7**.

**Table 7:** The top-20 lncRNAs predicted for prostate cancer.

Rank	LncRNA	Pubmed ID	Rank	LncRNA	Pubmed ID
1	H19	24988946	11	IGF2-AS	27507663
2	HOTAIR	23936419	12	PCAT1	22664915
3	MEG3	14602737	13	LincRNA-p21	27976428
4	MALAT1	23845456	14	PTENpg1	not found
5	CDKN2B-AS1	20541999	15	PRNCR1	20874843
6	PVT1	23728290	16	SNHG16	not found
7	GAS5	22664915	17	MINA	not found
8	Linc00963	24691949	18	SRA1	16607388
9	C1QTNF9B-AS1	27507663	19	NEAT1	25415230
10	UCA1	27686228	20	LSINCT5	not found

Colon cancer is considered as one of the most widespread and deadly cancers in the world. Disorders of lncRNAs are associated with miscellaneous biological processes, including tumorigenesis (Ba-Alawi et al., 2016). For example, CDKN2B-AS1 up-regulates proliferation in HCT116 cells in a manner independent of the p15/p16-pRB pathway (Chiyomaru et al., 2013). The lower expression of GAS5 is highly related to big tumor volumes, low histological scores and late TNM stages. LncRNA Plasma UCA1 can be used as a potential biomarker for inchoate diagnosis and monitoring of colon cancer (Aken et al., 2016). It is found that overexpression of lncRNA TUG1 promotes colon cancer progression (Ba-Alawi et al., 2016). We utilize FRMCLDA to restore the possible colon cancer-correlated lncRNAs. The results suggest that, 9 out of the top 10 (9/10) and 15 out of the top 20 (15/20) predicted lncRNAs are confirmed by three databases mentioned before (LncRNADisease, MNDR, Lnc2Cancer), as shown in **Table 8**.

**Table 8:** The top-20 lncRNAs predicted for colon cancer.

Rank	Name of LncRNA	Pubmed ID	Rank	Name of LncRNA	Pubmed ID
1	CDKN2B-AS1	26708220	11	DRAIC	Not found
2	PVT1	25043044	12	IGF2-AS	Not found
3	GAS5	25326054	13	NPTN-IT1	23395002
4	LincRNA-p21	26656491	14	XIST	29679755
5	UCA1	26885155	15	PCAT29	Not found
6	KCNQ1OT1	16965397	16	LSINCT5	25526476
7	TUG1	27634385	17	anti-NOS2A	Not found
8	MINA	Not found	18	HIF1A-AS2	29278853
9	BCYRN1	29625226	19	SNHG16	24519959
10	MIAT	29686537	20	HIF1A-AS1	28946548

Gastric cancer is one of the cancers with the highest incidence and mortality in the world. Gastric cancer is a complicated disease, caused by an imbalance of the cancer-causing and cancer-suppressing pathways (Aken et al., 2016). An increasing number of studies show that lncRNAs may play an active role in primary processes of gastric cancer. FRMCLDA predicts the gastric cancer-associated lncRNAs, some of which are validated though the latest public literature and databases. For instance, the expression of GAS5 is found to be lowered in gastric tumors, contrary to the up-regulated expression of mir-23a (Ba-Alawi et al., 2016). It is suggested that a high level of MALAT1 could be a potential biomarker for distant metastasis of gastric cancer. Further studies have shown that lincrna-p21 knockout can promote the malignant behavior of gastric cancer cells according to overexpression assay (Aken et al., 2016). LncRNA SNHG16 is found to be highly expressed in gastric cancer and thus has become a novel target of clinical treatment for gastric cancer (Lian et al., 2017). The results show that, 8 out of the top-10 ranked lncRNAs and 16 out of the top-20 ranked lncRNAs are validated by FRMCLDA, as shown in **Table 9**.

**Table 9:** The top-20 lncRNAs predicted for gastric cancer.

Rank	Name of LncRNA	Pubmed ID	Rank	Name of LncRNA	Pubmed ID
1	GAS5	27827524	11	SNHG16	29081409
2	MALAT1	27486823	12	PTENpg1	25694351
3	LincRNA-p21	28969031	13	PCAT29	25700553
4	BCYRN1	29435146	14	XIST	29053187
5	KCNQ1OT1	Not found	15	BDNF-AS1	Not found
6	IGF2-AS	Not found	16	HIF1A-AS1	26722487
7	TUG1	27983921	17	HIF1A-AS2	25686741
8	NPTN-IT1	28951520	18	lncRNA-ATB	28115163
9	MIAT	29039602	19	HAR1B	Not found
10	DRAIC	25700553	20	CCAT2	29435046

**Figure 6:**
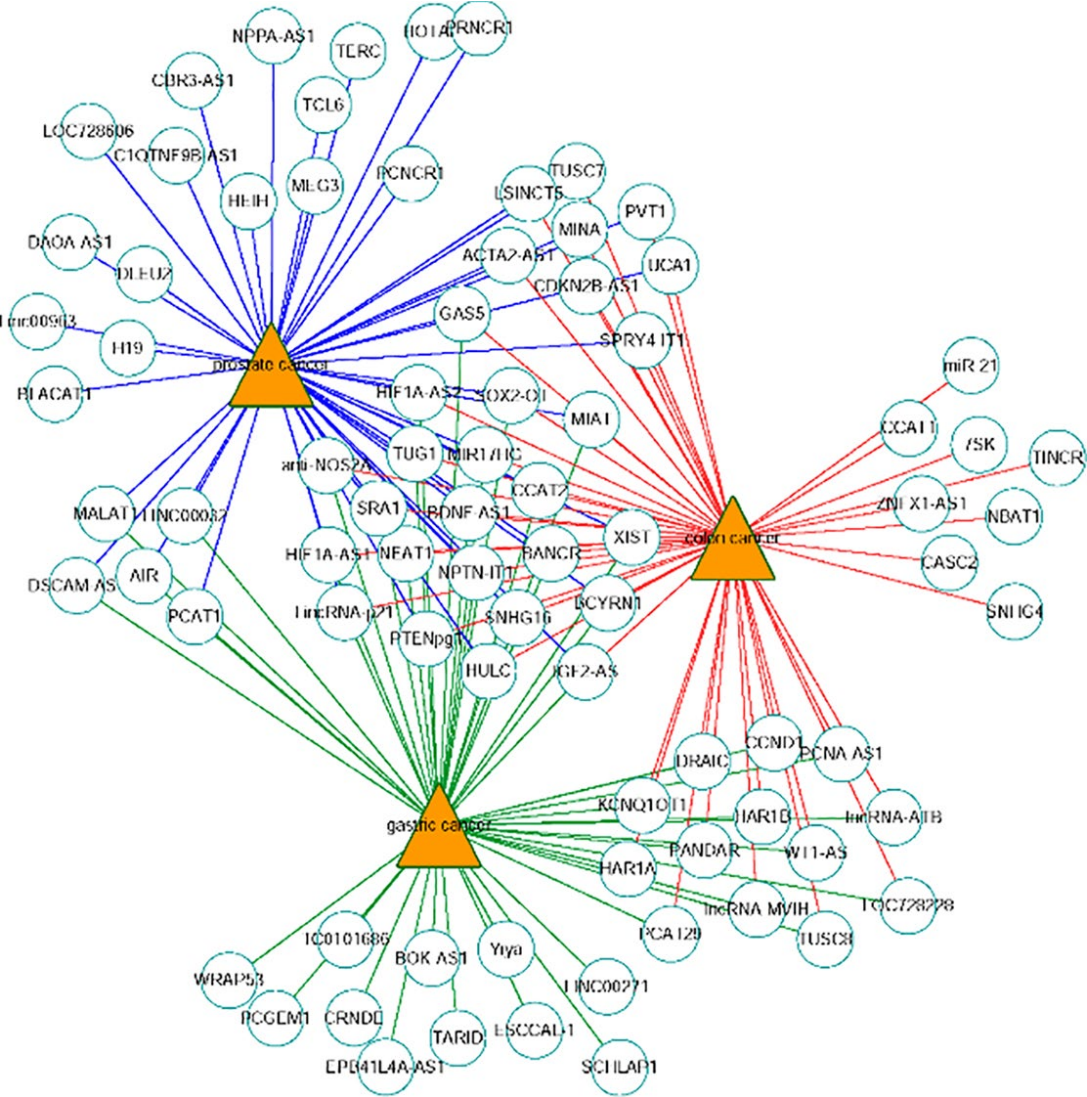
Network of the top-50 predicted associations of prostate cancer, colon cancer and gastric cancer on Dataset 3. Circles and triangles represent lncRNAs and diseases, respectively.

The network of the top 50 ranked links with prostate cancer, colon cancer and gastric cancer on Dataset 3 by FRMCLDA is shown in **Figure 6**. We find that some top-ranked lncRNAs are associated with one or more diseases. The results in case studies for three selected cancers have shown an outstanding prediction performance of FRMCLDA. As stated, FRMCLDA is a comprehensive method which could infer latent disease-lncRNA link for overall diseases synchronously. As a result, we also prioritized overall candidate disease-lncRNA pairs (excluding known links) on Dataset 3 by their global scores assigned through FRMCLDA. The higher the global scores of the links, the more likely that links between them exist. For example, the predicted global score for GAS5 and gastric cancer ranks 6th out of all the 60,169 non-zero predicted results by FRMCLDA. This prediction was confirmed in the latest research by Sun M et al. (Aken et al., 2016). They verified that the reduced expression of GAS5 indicates poor prognoses and will lead to gastric cancer cells spreading. Therefore, top-priority prediction further proves the validity of FRMCLDA, and so do the other high-ranked links. The results of the global rank for all the predicted links are provided in **Supplementary S3**. We hope that the prediction results may help discovery of disease-related lncRNAs.

## Conclusions

With the development of the next-generation sequencing in biomedical research, constructing a heterogeneous network on the basis of clinical NGS big data will benefit in prediction models of latent human disease-lncRNA associations. The prediction of disease-lncRNA links is very important in the biomedical field, among others. Construction of computational prediction models for new disease-lncRNA relationships will help understand the molecular mechanism of complicated human diseases at the level of lncRNA, and recognize the disease biomarker for diagnosis, treatment, prognosis and prevention of disease.

In this paper, we calculated the integrated similarities for diseases and lncRNAs using different methods and dealing with different types of data sources. We constructed a heterogeneous bilayer network by integrating similarity networks and interaction network. Then we utilized the algorithm fSVT to retrieve the unknown entries in adjacency matrix of the heterogeneous network. Theoretically FRMCLDA has a superior performance compared to other association prediction methods, because it takes account of all the predominant eigenvalues and the relevant eigenvectors of the matrix to be restored. In addition, FRMCLDA is able to process large scale matrices and execute proximate SVD rapidly at each SVT iteration by incorporating rSVD-BKI with a novel sub-space reuse technique. To assess the performances of FRMCLDA, experiments including global LOOCV and local LOOCV, global 5-fold CV and case studies are conducted. The experimental results show that the effectiveness of FRMCLDA is consistent with the theoretical estimation. Nevertheless, there are also a few limitations for FRMCLDA. First, if the adjacency matrix lacks low rank, then matrix completion with fSVT will lose its speed advantage. Second, the p value in power iteration can be adapted to guarantee the accuracy of SVD, but it can increase to several tens in some situations, which will lead to an increased running time and deprive the rSVD-BKI of advantages over the original SVD method. In conclusion, by expediting the matrix completion algorithm or properly extracting more effective features from lncRNAs and diseases, the performances of FRMCLDA can be further improved. 

## Data Availability

All datasets generated for this study are included in the manuscript/supplementary files.

## Author Contributions

WL, SW and JY conceptualized the work and planned the procedure of experiments; JX and GM and GT implemented literature research; WL collected the data and analysed the results; WL and JY drafted the manuscript; all the authors have read and supported the final edition.

## Funding

This work was supported by the National Nature Science Foundation of China (Grant Nos. 61672011 and 61474267, the National Key Research and Development Program (Grant Nos. 2017YFC1311003) and the Natural Science Foundation of Hunan, China (Grant No. 2018JJ2461).

## Conflict of Interest Statement

JY and GT were employed by company Geneis Beijing Co., Ltd. The remaining authors declare that the research was conducted in the absence of any commercial or financial relationships that could be construed as potential conflict of interest.
