# Supplementary material for: Inferring Latent Disease-lncRNA Associations by Faster Matrix Completion on a Heterogeneous Network
Source: Front Genet. 2019 Sep 4;10:769. doi: 10.3389/fgene.2019.00769 (PMC6749816; doi:10.3389/fgene.2019.00769)
Supplement: Supplementary file 6 [file Table_6.docx]

Supplementary Material

**Inferring Latent Disease-lncRNA Associations by Faster Matrix**

**Completion on a Heterogeneous Network**

**Wen Li^1^, Shulin Wang^1*^, Junlin Xu^1^, Guo Mao^1^, Geng Tian^2^, Jialiang Yang ^2*^**

^1^ College of Information Science and Engineering, Hunan University, Changsha, Hunan, 410082, P.R. China

^2^ Geneis Beijing Co., Ltd., Beijing, 100102, China

*** Correspondence:**

Shulin Wang: smartforesting@163.com

Jialiang Yang: yangjl@geneis.cn

Supplementary Algorithm

| Algorithm 1 : FRMCLDA algorithm using faster SVT |
| --- |
| **Input:** lncRNAs similarity matrix *LS*, disease similarity matrix *DS* and incomplete lncRNA-disease association matrix *LD.* Sampling set *Φ*, step size *δ* , the error limit *ε* , Singular value threshold *τ* , increment *l*, max iteration number *i*_max_.  **Output:** Completed lncRNA-disease association matrix *LD**   1. *Calculate lncRNA integrate similarity LS and disease integrate similarity matrix DS based on the association matrix LD.* 2. *Construct a large sparse adjacent matrix A for heterogeneous network.* 3. Initiation: $Y^{0}=c\delta P_{\Phi}(M)$, $c=\left\lceil\tau/(\delta\left\Vert P_{\Phi}\left( A \right) \right\Vert) \right\rceil$, *r0 = 0*, *q = 0*, *p = 2*, *l*=5, *i_max_*=1000. 4. **for** *i = 1,2, · · ·, i_max_* **do** 5. *k_i_ = r_i-1_ + l, then regulate the value of p dynamically* 6. **repeat** 7. **if** *i < i_reuse_* ***or*** *q == q_reuse_* ***then*** 8.  9. *q = 0* 10. ***else*** 11. *reuse Q in last round execution of rSVD-BKIr algorithm, and then compute , , * 12. *q = q + 1;* 13. ***end if*** 14. ** 15. ***until *** 16. ** 17. * /*linearized Bregmans interations*/* 18. ***if*** *$\left\Vert P_{\Phi}\left(X^{(i)}\right)-P_{\Phi}\left(M\right)\right\Vert_{F}/\left\Vert P_{\Phi}\left(M\right)\right\Vert_{\text{F }}<\varepsilon$* ***then break*** 19. ** 20. ***end for*** 21. ** 22. ***Return*** *LD^*^* |

%% where the rSVD-BKI is referenced from the literature: “Faster Matrix Completion Using Randomized SVD”
