# Supplementary material for: Inferring Latent Disease-lncRNA Associations by Faster Matrix Completion on a Heterogeneous Network
Source: Front Genet. 2019 Sep 4;10:769. doi: 10.3389/fgene.2019.00769 (PMC6749816; doi:10.3389/fgene.2019.00769)
Supplement: Supplementary file 7 [file Table_7.docx]

**Codes**

%%% main %%

clear;

clc;

for k=1:1

k;

globalposition=FRMCLDA_LOOCV_DATA1();

overallauc1(k)=positiontooverallauc_loocv_DATA1();

%

% globalposition=FRMCLDA_LOOCV_DATA2();

% overallauc2(k)=positiontooverallauc_loocv_DATA2();

%

% globalposition=FRMCLDA_LOOCV_DATA3();

% overallauc3(k)=positiontooverallauc_loocv_DATA3();

end

save FRMCLDA_LOOCV_DATA1_COSINE;

% a1=mean(overallauc1); % mean of auc values

% b1=std(overallauc1); % standard deviation of auc values

%%%%%%%%%%%%%%%%%%%%%%%%%%%%%%%%%%%%%%%%%%%%%%%%%%%%%%%

function [ result ] = cosSim( data )

%COSSIM Summary of this function goes here

% Detailed explanation goes here

rows=size(data,1);

result=zeros(rows,rows);

for i=1:rows

for j=1:i

if (norm(data(i,:))*norm(data(j,:))==0)

result(i,j)=0;

else

result(i,j)=dot(data(i,:),data(j,:))/(norm(data(i,:))*norm(data(j,:)));

%%n=norm(A) % Returns the maximum singular value of A

%%dot(a,b) % he dot product of vectors a and b

end

result(j,i)=result(i,j); %% symmetric matrix

end

end

%%%%%%%%%%%%%%%%%%%%%%%%%%%%%%%%%%%%%%%%%%%%%%%%%%%%

function globalposition=FRMCLDA_LOOCV_DATA1()

load data1

[m1,n2]=size(LD);

Adj=[LS,LD;LD',DS];

% color block matrix

% figure(1);

% image(Adj,'CDataMapping','scaled');

% colorbar;

interaction=LD;

save interaction1 interaction;

index=find(LD);

[pp,qq]=size(index);

A=zeros(pp,2);

for i=1:pp

[lncrna,disease]=ind2sub(size(LD),index(i));

A(i,1)=lncrna;

A(i,2)=disease;

end

save('knownlinks1.mat','A');

%implement leave-one-out cross validation

for cv=1:pp

cv

% obtain training sample

load interaction1;

interaction(A(cv,1),A(cv,2))=0;

% %%%%%%%%%% similarity computation %%%%%%%%%%%%%%%%%%%

w1=0.7;

w2=0.9;

Slcos = cosSim(interaction);

Sdcos = cosSim(interaction');

LS=w1*LS+(1-w1)*Slcos;

DS=w2*DS+(1-w2)*Sdcos;

%%%%%%%%% matrix completion %%%%%%%%%%%%%%%%%%%%%%%%%%

Adj1=[LS,interaction;interaction',DS];

SP=sparsity(Adj1)

M = sparse(Adj1);

t = cputime;

[X] = fastSVT_Q(M, 0.4, [0 1], 100, 10); %%tol=0.4, ran=[0,1], r_reuse=100,q_reuse=10

t_SVT = cputime - t;

F=X(1:m1,(m1+1):(m1+n2)); % F is the matrix of predicted scores

finalscore=F(A(cv,1),A(cv,2));

for i=1:m1

for j=1:n2

if interaction(i,j)==1

F(i,j)=-10000;

end

end

end

% obtain the position of tested disease-microbe interaction as variable globalposition(1,cv),

[ll1,mm1]=size(find(F>=finalscore));

[ll2,mm2]=size(find(F>finalscore));

globalposition(1,cv)=ll2+1+(ll1-ll2-1)/2;

end

save('globalposition_LOOCV_DATA1_COSINE.mat','globalposition');

end

%%%%%%%%%%%%%%%%%%%%%%%%%%%%%%%%%%%%%%%%%%%%%%%%%%%%

function overallauc=positiontooverallauc_loocv_DATA1()

load globalposition_LOOCV_DATA1_COSINE.mat;

load interaction1;

[n,m]=size(interaction);

sID=importdata('knownlinks1.mat');

[pp,qq]=size(sID);

for i=1:pp

if globalposition(i)>m*n-pp+1

globalposition(i)=m*n-pp+1;

end

end

pre_y=[];

rec_y=[];

for k=1:m*n-pp+1

tp=0;

for t=1:pp

if globalposition(1,t)<=k

tp=tp+1;

end

end

tpr(1,k)=tp/pp;

fp=k*pp-tp;

fpr(1,k)=fp/(pp*(m*n-pp));

fn=pp-tp;

Precision(1,k)=tp/(k*pp);

recall(1,k)=tp/(tp+fn);

if mod(k,10)==0

pre_y(1,k)=Precision(1,k);

rec_y(1,k)=recall(1,k); %calculate the recall

end

%%%%%%%%%%%%%%%%%%%%%%%%%%%%%%%%%%%%%%%%%%%%%%%%%%%%%%%%%%%%%%%%%%%%%%%%

end

Spe=1-fpr;

Sen=recall;

AUC=abs(trapz(1-Spe,Sen));

AUPR=abs(trapz(Sen,Precision));

overallauc=AUC;

% plot(fpr,tpr);

% str = ['AUC= ',num2str(AUC),'AUPR=',num2str(AUPR)];

% title('FMCLDA ROC curve');

% text(0.3,0.2,str);

% axis([-0.01 1.00 0 1.01]);

% xlabel('FPR');

% ylabel('TPR');

% figure;

% plot(recall,Precision);

% %%%%%%%%%%%%%%%%%%%%%%%%%%%%%%%%%%%%%%%%%%%%%%%%%%%%%%%%%%%%%%%%%%%%%%%%%

% figure;

% bar(pre_y,5);

% xlabel('top-k');

% ylabel('precision');

% title('FMCLDA precision-rank');

% axis([0 300 0 0.02]);

% set(gca,'XTickLabel',{'','','top-20','','top-40','','top-60','','top-80','','top-100'})

% figure;

% bar(rec_y,5);

% xlabel('top-k')

% ylabel('recall')

% title('FMCLDA recall-rank');

% axis([0 300 0 0.5]);

% set(gca,'XTickLabel',{'','top-20','','top-40','','top-60','','top-80','','top-100'})

end
